# Supplementary material for: A tunable transition metal dichalcogenide entangled photon-pair source
Source: Nat Commun. 2024 Sep 1;15:7600. doi: 10.1038/s41467-024-51843-3 (PMC11366010; doi:10.1038/s41467-024-51843-3)
Supplement: Supplementary file 1 — Supplementary Information [file 41467_2024_51843_MOESM1_ESM.pdf]

Supplementary Information:
A Tunable Transition Metal Dichalcogenide Entangled Photon-Pair Source

Maximilian A. Weissflog,[1,2,*] Anna Fedotova,[1,3] Yilin Tang,[4] Elkin A. Santos,[1]
Benjamin Laudert,[1] Saniya Shinde,[1] Fatemeh Abtahi,[1] Mina Afsharnia,[1] Inmaculada Pérez Pérez,[1]
Sebastian Ritter,[1] Hao Qin,[4] Jiri Janousek,[4,5] Sai Shradha,[1,6] Isabelle Staude,[1,3] Sina Saravi,[1]
Thomas Pertsch,[1,7] Frank Setzpfandt,[1,7] Yuerui Lu,[4,5,†] and Falk Eilenberger[1,7,‡]

[1]*Institute of Applied Physics, Abbe Center of Photonics,*
Friedrich Schiller University Jena, Albert-Einstein-Straße 15, Jena, 07745, Germany
[2]*Max Planck School of Photonics, Hans-Knöll-Straße 1, Jena, 07745, Germany*
[3]*Institute of Solid State Physics, Friedrich Schiller University Jena, Helmholtzweg 3, Jena, 07743, Germany*
[4]*School of Engineering, College of Science and Computer Science,*
The Australian National University, Canberra, Australian Capital Territory, Australia
[5]*Australian Research Council Centre of Excellence for Quantum Computation and Communication Technology,*
The Australian National University, Canberra, Australian Capital Territory, Australia
[6]*Institute for Condensed Matter Physics, Technical University of Darmstadt,*
Hochschulstraße. 6-8, Darmstadt, 64289, Germany
[7]*Fraunhofer Institute for Applied Optics and Precision Engineering IOF,*
Albert-Einstein-Straße 7, Jena, 07745, Germany

CONTENTS

Supplementary Note 1: Concurrence, State Fidelity and Theoretical SPDC Rate for Measurements with a Single Analyzer 2

Supplementary Note 2: Influence of Out-of-plane Nonlinear Tensor Components on Generated Quantum State 5

Supplementary Note 3: Thickness Dependent SHG Measurements 7

Supplementary Note 4: Details of Experimental Setup for Photon-Pair Measurements 8

Supplementary Note 5: Raw Data Coincidence Measurements 10
A. Power Dependence Test 10
B. Polarization Analysis 11
C. Quantum-State Tomography 12

References 15

* maximilian.weissflog@uni-jena.de
† yuerui.lu@anu.edu.au
‡ falk.eilenberger@uni-jena.de

SUPPLEMENTARY NOTE 1: CONCURRENCE, STATE FIDELITY AND THEORETICAL SPDC RATE FOR MEASUREMENTS WITH A SINGLE ANALYZER

In this section we derive the analytical dependence of the concurrence C and the state fidelity of the polarization quantum state $|\psi\rangle$ on the pump polarization angle, as shown in Fig. 1(c) of the main text. Furthermore, we provide a short derivation of the theoretical dependence of the photon-pair rate for measurements with a single analyzer for signal and idler photons. This corresponds to the theoretical curves in Fig. 3 of the main text.

We start from the general polarization quantum state generated by a transition metal dichalcogenide for a linearly polarized excitation propagating along the z-axis. Given a nonlinear tensor with the components $\chi^{(2)}_{yyy} = -\chi^{(2)}_{yxx} = -\chi^{(2)}_{xxy} = -\chi^{(2)}_{xyx}$ the quantum state reads

$$|\psi\rangle = \frac{\sin(\varphi_p)}{\sqrt{2}}\left(|\mathrm{HH}\rangle - |\mathrm{VV}\rangle\right) + \frac{\cos(\varphi_p)}{\sqrt{2}}\left(|\mathrm{HV}\rangle + |\mathrm{VH}\rangle\right), \tag{1}$$

which corresponds to Eq. 1 of the main text. Here, φ_p is the polarization angle of the pump photon as defined in Fig. 1(b) of the main manuscript.

As an entanglement measure, we employ the state concurrence C, which is $C = 0$ for separable states, monotonically increases with the degree of entanglement reaches $C = 1$ for maximally entangled states [1]. In the following we choose a description using density matrices, since the same formalism for calculating concurrence and state fidelites can then be directly applied to the density matrices measured in the experiment.

We first calculate the related density matrix $\hat{\rho}$

$$\hat{\rho} = |\psi\rangle\langle\psi| = \frac{1}{2}\begin{pmatrix} \sin^2(\varphi_\mathrm{p}) & \sin(\varphi_\mathrm{p})\cos(\varphi_\mathrm{p}) & \sin(\varphi_\mathrm{p})\cos(\varphi_\mathrm{p}) & -\sin^2(\varphi_\mathrm{p}) \\ \sin(\varphi_\mathrm{p})\cos(\varphi_\mathrm{p}) & \cos^2(\varphi_\mathrm{p}) & \cos^2(\varphi_\mathrm{p}) & -\sin(\varphi_\mathrm{p})\cos(\varphi_\mathrm{p}) \\ \sin(\varphi_\mathrm{p})\cos(\varphi_\mathrm{p}) & \cos^2(\varphi_\mathrm{p}) & \cos^2(\varphi_\mathrm{p}) & -\sin(\varphi_\mathrm{p})\cos(\varphi_\mathrm{p}) \\ -\sin^2(\varphi_\mathrm{p}) & -\sin(\varphi_\mathrm{p})\cos(\varphi_\mathrm{p}) & -\sin(\varphi_\mathrm{p})\cos(\varphi_\mathrm{p}) & \sin^2(\varphi_\mathrm{p}) \end{pmatrix}. \tag{2}$$

Together with the "spin flip" matrix $\hat{\Sigma}$

$$\hat{\Sigma} = \begin{pmatrix} 0 & 0 & 0 & -1 \\ 0 & 0 & 1 & 0 \\ 0 & 1 & 0 & 0 \\ -1 & 0 & 0 & 0 \end{pmatrix} \tag{3}$$

we can then define the non-Hermitian matrix $\hat{R} = \hat{\rho}\hat{\Sigma}\hat{\rho}^T\hat{\Sigma}$ [2]. When labeling the eigenvalues of $\hat{R}$ in decreasing order such that $r_1 \geq r_2 \geq r_3 \geq r_4$, the concurrence C follows from [2]

$$C = \max\left\{0, \sqrt{r_1} - \sqrt{r_2} - \sqrt{r_3} - \sqrt{r_4}\right\}. \tag{4}$$

For the quantum state $|\psi\rangle$ generated by the TMD, we find

$$\hat{R} = \frac{1}{2}\begin{pmatrix} \sin^2(\varphi_\mathrm{p}) & \frac{1}{2}\sin(2\varphi_\mathrm{p}) & \frac{1}{2}\sin(2\varphi_\mathrm{p}) & -\sin^2(\varphi_\mathrm{p}) \\ \frac{1}{2}\sin(2\varphi_\mathrm{p}) & 1-\sin^2(\varphi_\mathrm{p}) & 1-\sin^2(\varphi_\mathrm{p}) & -\frac{1}{2}\sin(2\varphi_\mathrm{p}) \\ \frac{1}{2}\sin(2\varphi_\mathrm{p}) & 1-\sin^2(\varphi_\mathrm{p}) & 1-\sin^2(\varphi_\mathrm{p}) & -\frac{1}{2}\sin(2\varphi_\mathrm{p}) \\ -\sin^2(\varphi_\mathrm{p}) & -\frac{1}{2}\sin(2\varphi_\mathrm{p}) & -\frac{1}{2}\sin(2\varphi_\mathrm{p}) & \sin^2(\varphi_\mathrm{p}) \end{pmatrix} \tag{5}$$

and as a result a concurrence of

$$C = \left(\cos^4(\varphi_\mathrm{p}) + \sin^4(\varphi_\mathrm{p}) + 2\cos^2(\varphi_\mathrm{p})\sin^2(\varphi_\mathrm{p})\right)^{1/2} = 1. \tag{6}$$

This shows that $C = 1$ independent of the linear polarization angle φ_p, meaning that any of the polarization states generated by the TMD is maximally entangled.

The fidelity of a quantum state with density matrix $\hat{\rho}$ to a second quantum state with density matrix $\hat{\sigma}$ is defined as [3]

$$F(\hat{\rho},\hat{\sigma}) = \left(Tr\left(\sqrt{\sqrt{\hat{\rho}}\,\hat{\sigma}\sqrt{\hat{\rho}}}\right)\right)^2 . \tag{7}$$

For two pure quantum states with density matrices $\hat{\rho} = |\psi_\rho\rangle\langle\psi_\rho|$ and $\hat{\sigma} = |\psi_\sigma\rangle\langle\psi_\sigma|$, Eq. (7) simplifies to

$$F(\hat{\rho},\hat{\sigma}) = \left|\langle\psi_\rho|\psi_\sigma\rangle\right|^2 . \tag{8}$$

From here it is straightforward to see, that the fidelity of the polarization state generated by the TMD Eq. (1) with the Bell state $|\Psi^+\rangle = \frac{1}{\sqrt{2}}\left(|\mathrm{HV}\rangle + |\mathrm{VH}\rangle\right)$ varies with the pump polarization angle φ_p as

$$F(\psi(\varphi_\mathrm{p}),\Psi^+) = \left|\langle\psi(\varphi_\mathrm{p})|\Psi^+\rangle\right|^2 = \cos^2(\varphi_\mathrm{p}). \tag{9}$$

The fidelity with the Bell state $|\Phi^-\rangle = \frac{1}{\sqrt{2}}\left(|\mathrm{HH}\rangle - |\mathrm{VV}\rangle\right)$ is

$$F(\psi(\varphi_\mathrm{p}),\Phi^-) = \left|\langle\psi(\varphi_\mathrm{p})|\Phi^-\rangle\right|^2 = \sin^2(\varphi_\mathrm{p}). \tag{10}$$

The here derived dependencies for the concurrence Eq. (6), and the state fidelities Eq. (9) and (10), respectively, are visualized in Fig. 1(c) of the main manuscript.

We now turn to the calculation of the SPDC rate for detection through a single analyzer for signal and idler photon as reported in Fig. 3 of the main manuscript. First, we define the polarization state of a single, linearly polarized photon with polarization angle φ_pol, which reads

$$|\psi_\mathrm{pol}\rangle = \cos\left(\varphi_\mathrm{pol}\right)|\mathrm{H}\rangle + \sin\left(\varphi_\mathrm{pol}\right)|\mathrm{V}\rangle . \tag{11}$$

The operator $P(\varphi_\mathrm{pol})$ for a projection of a single photon passing through a polarizer with orientation along φ_pol follows from

$$P(\varphi_\mathrm{pol}) = |\psi_\mathrm{pol}\rangle\langle\psi_\mathrm{pol}| . \tag{12}$$

The simultaneous measurement of signal and idler through the same polarizer corresponds to the projector $P_\mathrm{si}(\varphi_\mathrm{pol})$

$$P_\mathrm{si}(\varphi_\mathrm{pol}) = P(\varphi_\mathrm{pol}) \otimes P(\varphi_\mathrm{pol}). \tag{13}$$

The normalized coincidence rate $R_\mathrm{SPDC}(\varphi_\mathrm{p},\varphi_\mathrm{pol})$ depending on pump polarization angle and polarizer angle is then

$$R_\mathrm{SPDC}(\varphi_\mathrm{p},\varphi_\mathrm{pol}) = \langle\psi|\,P_\mathrm{si}(\varphi_\mathrm{pol})\,|\psi\rangle = \frac{1}{2}\sin^2(2\varphi_\mathrm{pol} + \varphi_\mathrm{p}). \tag{14}$$

This is a function periodic in φ_p with period π and periodic in φ_pol with period $\pi/2$. To visualize this, we plot eq. (14) in Supplementary Fig. 1. The measurements reported in Fig. 3 of the main manuscript correspond to 'cut-lines' that sample the two-variable function for different configurations. The measurements with rotating pump and polarizer in parallel or perpendicular configuration (Fig. 3(c) of the main text) are marked with red and gray dashed lines, respectively. The configurations with fixed analyzer and rotating pump (Fig. 3(d)-(f) of the main text), are highlighted with green dashed lines in Supplementary Fig. 1. The very good agreement between the predicted and experimentally measured functional dependence confirms eq. (14).

Based on this, also the dependence for an experimental setting that we didn't explore, namely fixed pump polarization and rotating polarizer, can be evaluated. This would correspond to horizontal 'cut-lines' in Supplementary Fig. 1. This leads to a characteristic four-lobed pattern of the SPDC rate, which we visualize in Supplementary Fig. 2.

Lastly, we take a closer look at the importance of the reference angle in case of co-rotating pump polarization and analyzer angle.

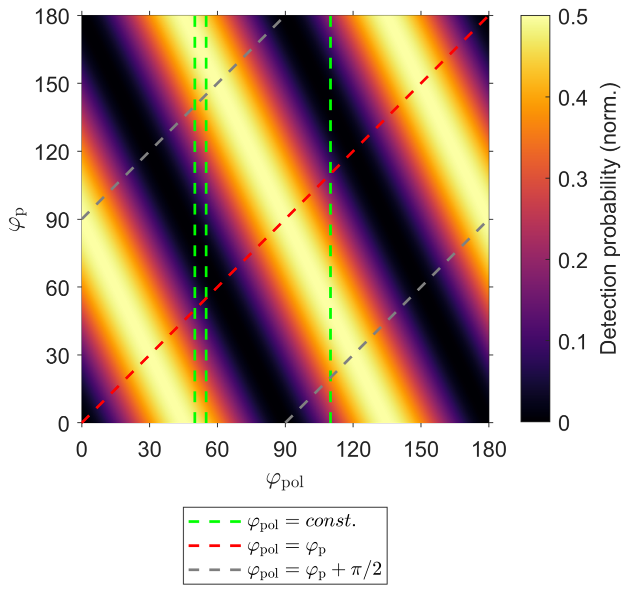

Supplementary Fig. 1. SPDC rate measured through single analyzer depending on analyzer angle φ_{pol} and pump polarization angle φ_{p}. Visualization of the periodic function eq. (14). The experimental configurations corresponding to the measurement results in Fig. 3 of the main manuscript are marked with coloured, dashed lines: parallely co-rotating pump and polarizer (red), perpendicularly co-rotating pump and polarizer (gray), fixed polarizer and rotating pump (green).

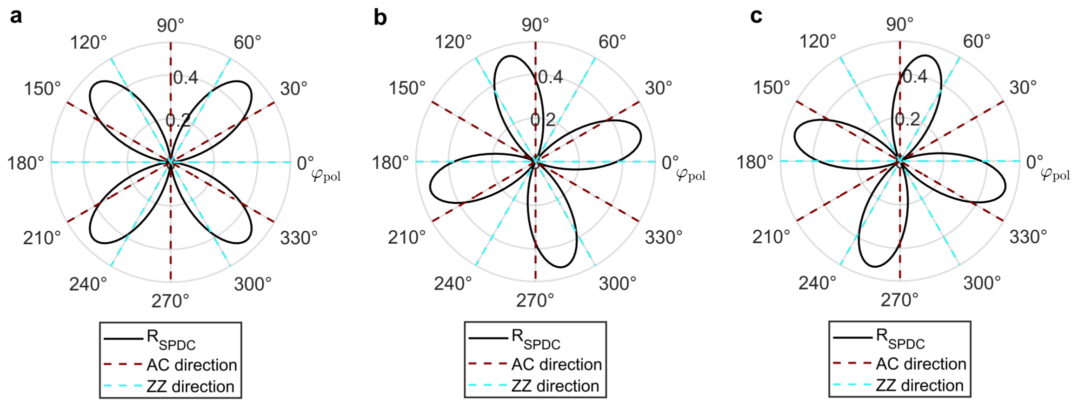

Supplementary Fig. 2. SPDC rate for fixed pump polarization and rotating analyzer polarization. a Theoretical dependence of SPDC rate measured through a common polarizer for signal and idler photons for a rotating analyzer and a pump polarization angle fixed at a) $\varphi_{\mathrm{p}} = 0°$, b) $\varphi_{\mathrm{p}} = 60°$ and c) $\varphi_{\mathrm{p}} = 120°$, respectively.

From eq. (14) it is straightforward to see that for an alignment of the pump polarization either in parallel $R_{\mathrm{SPDC},\parallel}$ ($\varphi_{\mathrm{pol}} = \varphi_{\mathrm{p}}$) or in orthogonal configuration $R_{\mathrm{SPDC},\perp}$ ($\varphi_{\mathrm{pol}} = \varphi_{\mathrm{p}} \pm \pi/2$), the dependence is $R_{\mathrm{SPDC},\parallel} = 1/2\sin^2\left(2\varphi_{\mathrm{p}} + \varphi_{\mathrm{p}}\right) = R_{\mathrm{SPDC},\perp} = 1/2\sin^2\left(2(\varphi_{\mathrm{p}} \pm \pi/2) + \varphi_{\mathrm{p}}\right) = 1/2\sin^2\left(3\varphi_{\mathrm{p}}\right)$. This curve is shown in Supplementary Fig. 3(a) and matches the experimental results as shown in Fig. 3(c) of the main text. We note here, that the characteristic six-fold pattern of the SPDC rate is co-aligned for both parallel and perpendicular analyzer configuration, when using the polarization angle of the SPDC pump photon φ_{p} as reference. This is in fact in full agreement with measurements of polarized SHG from TMDs, that reported a shift of 30° of the 6-lobed patterns obtained for parallel or perpendicular excitation [4]. To illustrate this, we also look at the case where the rotation angle of the analyzer φ_{pol} is used as reference. Then we find for the parallel configuration with $\varphi_{\mathrm{p}} = \varphi_{\mathrm{pol}}$ the dependence $R_{\mathrm{SPDC},\parallel} = 1/2\sin^2\left(2\varphi_{\mathrm{pol}} + \varphi_{\mathrm{pol}}\right) = 1/2\sin^2\left(3\varphi_{\mathrm{pol}}\right)$. However, the perpendicular configuration where $\varphi_{\mathrm{p}} = \varphi_{\mathrm{pol}} \pm \pi/2$ yields $R_{\mathrm{SPDC},\perp} = 1/2\sin^2\left(2\varphi_{\mathrm{pol}} + \varphi_{\mathrm{pol}} \pm \pi/2\right) = 1/2\cos^2\left(3\varphi_{\mathrm{pol}}\right)$. Therefore, when referenced to the analyzer angle φ_{pol}, the two polarization patterns have a shift of 30°, see the plot in Supplementary Fig. 3(b).

In our SPDC measurement the angle of the analyzer φ_{pol} selects the polarization direction of the down-converted (low-energy) signal and idler photons. In a typical SHG measurement, these low-energy photons however act as the pump. Therefore, when directly comparing SPDC measurements with SHG measurements plotted in terms of the SHG pump angle, see e.g. our measurement reported in Fig. 3(a) of the main text, a direct equivalence is obtained when referencing the SPDC measurement to the analyzer angle φ_{pol}.

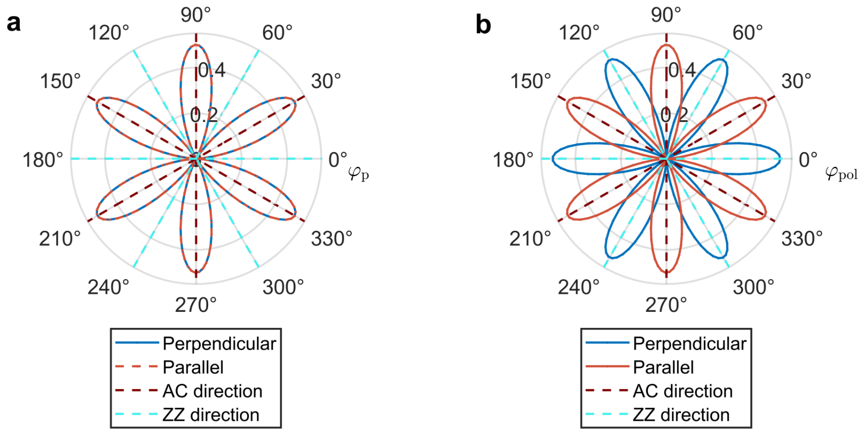

Supplementary Fig. 3. **SPDC rate for co-rotating pump polarization and analyzer polarization.** **a** Theoretical dependence of SPDC rate measured through a common polarizer for signal and idler photons oriented either perpendicularly or parallelly to the pump polarization. The dependence is plotted in terms of the pump polarization angle φ_{p}. This configuration is the same as chosen for the experiments in Fig. 3c of the main text. **b** The same theoretical dependence of polarized SPDC rate as in a), however now evaluated in terms of the analyzer polarization angle φ_{pol}. This configuration corresponds to the setting chosen for many polarized SHG measurements from TMDs [4].

SUPPLEMENTARY NOTE 2: INFLUENCE OF OUT-OF-PLANE NONLINEAR TENSOR COMPONENTS ON GENERATED QUANTUM STATE

Monolayer MoS_2 belongs to the point group D_{3h} with in-plane nonlinear tensor components $\chi^{(2)}_{\alpha\beta\gamma} = \chi^{(2)}_{yyy} = -\chi^{(2)}_{yxx} = -\chi^{(2)}_{xxy} = -\chi^{(2)}_{xyx}$ [5]. In the main text we have shown, that these tensor elements lead to an intrinsic creation of maximally entangled polarization quantum states. In the following we will show, that this property is not fundamentally changed by the additional nonlinear tensor elements of 3R-MoS_2.

The symmetry group of 3R-MoS_2 is C_{3v}, which compared to monolayer MoS_2, introduces the following additional nonlinear tensor elements: $\chi^{(2)}_{xzx} = \chi^{(2)}_{yzy}$, $\chi^{(2)}_{xxz} = \chi^{(2)}_{yyz}$, $\chi^{(2)}_{zxx} = \chi^{(2)}_{zyy}$ and $\chi^{(2)}_{zzz}$ [5]. Both the pump wavelength of $\lambda_p = 788\,\mathrm{nm}$ as well as the signal and idler wavelengths in the range $> 1300\,\mathrm{nm}$ are sufficiently far away from the excitonic resonances of 3R-MoS_2 which occur at $\lambda < 670\,\mathrm{nm}$ [6]. Therefore, the dispersion of $\chi^{(2)}$ in the frequency range of interest is negligible [6] and Kleinman's symmetry condition is valid. This leads to an equal magnitude of the tensor components $\chi^{(2)}_{xzx} = \chi^{(2)}_{yzy} = \chi^{(2)}_{xxz} = \chi^{(2)}_{yyz} = \chi^{(2)}_{zxx} = \chi^{(2)}_{zyy}$ [5]. In our convention, the index γ refers to the pump polarization direction. For a moderately focused Gaussian pump beam propagating along z, the z-polarized pump field components $E_z \approx 0$ are negligible. This means that the tensor elements with $\gamma = z$, $\chi^{(2)}_{xxz}$, $\chi^{(2)}_{yyz}$ and $\chi^{(2)}_{zzz}$, will practically not contribute to down-conversion. All other tensor elements involving $\alpha, \beta = z$ can generate a z-polarized signal or idler photon, which could potentially reduce the fidelity of the generated Bell or other entangled states.

We theoretically test the influence of these tensor components on the generation of entangled states using the Green's function (GF) method described in the methods section [7–10]. The important quantity for this is not the absolute nonlinearity value but the relative magnitude of the in-plane tensor components $|d_{16}| = 0.5\left|\chi^{(2)}_{xxy}\right|$ and the out-of-plane z-polarized tensor components $|d_{31}| = 0.5\left|\chi^{(2)}_{zxx}\right|$. For conversion from a fundamental wavelength of 1064 nm to a second-harmonic wavelength of 532 nm in 3R-MoS_2, this ratio b is measured in [11] as $b = \frac{|d_{16}|}{|d_{31}|} = \frac{500\,\mathrm{pm/V} \pm 200\,\mathrm{pm/V}}{200\,\mathrm{pm/V} \pm 100\,\mathrm{pm/V}} = 2.5 \pm 1.6$. It is now important to note that at these wavelengths $\chi^{(2)}$ of 3R-MoS_2 is already dispersive [6], which likely is the reason for the difference to the nonlinearity value of $d = 0.5\chi^{(2)} = 15\,\mathrm{pm/V}$ measured in [12] (fundamental wavelength 1200 nm, second-harmonic 600 nm).

Since we use different wavelengths outside of the dispersive region, these values will not apply exactly to our experiment. More importantly, next to the absolute nonlinearity value, also the ratio b of in- and out-of-plane components might change with wavelength. This needs to be taken into account in our analysis. Therefore, we consider the mean ratio of $b = 2.5$ measured in [11] only as a guideline and also test ratios that correspond to weaker ($b = 10$) or much stronger ($b = 0.25$) out-of-plane tensor components. Additionally, we also consider different collection numerical apertures of NA=0.4 (experimental case) and NA=0.9. For this set of parameters we compute the fidelity $F(\Phi^-)$ with the Bell state $\Phi^- = 1/\sqrt{2}\,(|HH\rangle - |VV\rangle)$ and the concurrence C for an x-polarized pump. The results are summarized in Supplementary Table 1.

Supplementary Table 1. Calculated state fidelities $F(\Phi^-)$ and concurrences C for different ratios b of in-plane and out-of-plane nonlinear tensor components and collection numerical apertures NA.

	$b = \lvert d_{16}\rvert / \lvert d_{31}\rvert$	NA=0.4	NA=0.9
$F(\Phi^-)$	$b = 10$	1	0.977
	$b = 2.5$	1	0.976
	$b = 0.25$	0.969	0.894
C	$b = 10$	1	0.954
	$b = 2.5$	1	0.894
	$b = 0.25$	0.964	0.814

As expected, we find as general trend that the state fidelity and concurrence decrease if the strength of the out-of-plane nonlinear tensor elements is increased. However, it is important to note that this effect is only pronounced if the collection numerical aperture is large. For a moderate numerical aperture of NA=0.4 as used in our experiment, the influence of the out-of-plane components is negligible for $b = 10$ and $b = 2.5$ and still small in case of $b = 0.25$. This is because for the out-of-plane tensor elements $\chi^{(2)}_{xzx}$, $\chi^{(2)}_{zxx}$, $\chi^{(2)}_{yzy}$ and $\chi^{(2)}_{zyy}$ either the signal or idler photon will be generated by an out-of-plane nonlinear source, leading to an emission under a large propagation angle. As a result, emission via these tensor elements can only be collected efficiently with large numerical apertures. Contrarily, the tensor elements that generate the entangled states $\chi^{(2)}_{yyy} = -\chi^{(2)}_{yxx} = -\chi^{(2)}_{xxy} = -\chi^{(2)}_{xyx}$ only have in-plane components, such that the emission is close to the optical axis. The difference is clearly seen in Supplementary Fig. 4, where we compare the calculated farfield emission patterns for the in-plane and out-of-plane components. While emission from the in-plane elements is concentrated around the z-axis (Supplementary Fig. 4(a)), the out-of-plane components have zero emission along the z-axis (Supplementary Fig. 4(b)).

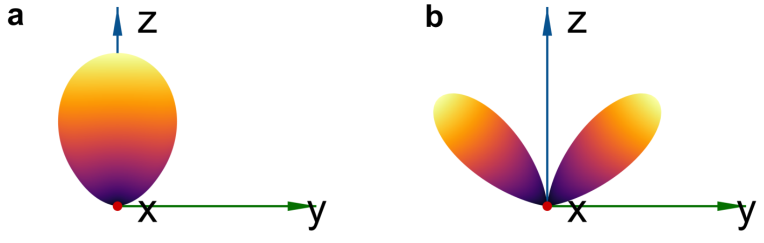

Supplementary Fig. 4. **Two-photon farfield emission pattern for in- and out-of-plane nonlinear tensor components.** Two-photon farfield emission pattern for (a) in-plane and (b) out-of-plane nonlinear tensor components for an x-polarized pump field. The two detectors for coincidence detection are scanned symmetrically around the z-axis [10]

This means that irrespective of the ratio between $|d_{16}|/|d_{31}|$, the quantum state emitted along the z-axis will always only be generated by in-plane tensor components. Since the relation of the in-plane elements $\chi^{(2)}_{yyy} = -\chi^{(2)}_{yxx} = -\chi^{(2)}_{xxy} = -\chi^{(2)}_{xyx}$ directly stems from the crystal symmetry, it is not subject to dispersion. With this, the quality of the entangled state generated by 3R-MoS_2 along the z-axis does not depend on the ratio of the in- and out-of-plane tensor components which could change with wavelength. This is true even in an extreme case, where the out-of-plane elements are nominally much stronger than the in-plane tensor elements. The degree of entanglement of photon pairs emitted close to the z-axis is the practically important configuration, since the use of longer crystals together with quasi-phasematching, cavities etc. will naturally lead to directed emission close to the z-axis. However, in this work we show experimentally, that also for collection with a moderate numerical aperture of NA=0.4 in practice a highly entangled state can be observed.

SUPPLEMENTARY NOTE 3: THICKNESS DEPENDENT SHG MEASUREMENTS

We compare the second-harmonic generation (SHG) efficiency of the 3R-MoS_2 crystal used for the SPDC experiments with a monolayer MoS_2 flake, as shown in Supplementary Fig. 5(a). For this measurement, we use an ultrafast laser (OPO Inspire Auto 100 pumped by Mai Tai HP, both Spectra-Physics) with pulse duration $\tau_p = 100$ fs and central wavelength 1550 nm, which is focused onto the sample with a 50x NIR objective (NA= 0.4, Mitutoyo). The signal is collected via an identical 50x NIR objective lens, then passed through an 800 nm short-pass filter (SP) to remove any fundamental beam component and detected on a spectrograph (Andor Kymera with iDuS 416-CCD). A confocal microscope above the first objective allows for sample imaging. We compare samples in the thickness range between the monolayers with $t \approx 0.7$ nm up to thicker crystals with $t = 285$ nm. Remarkably, the SHG signal from the 3R-MoS_2 crystal is about 1850 times stronger than the signal from the monolayer MoS_2.

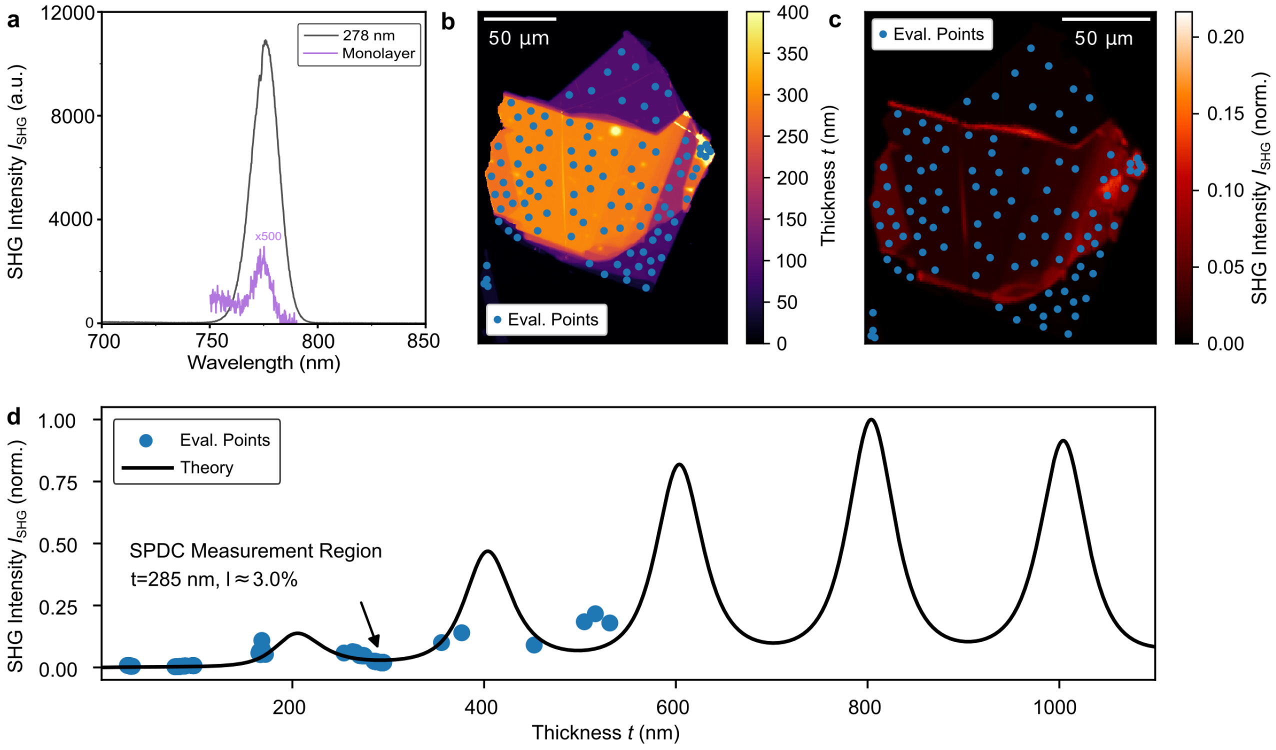

Supplementary Fig. 5. **Thickness-dependent second-harmonic generation efficiency of 3R-MoS_2. a** Comparison of SHG spectrum from a monolayer MoS_2 crystal and a thicker 3R-MoS_2 crystal. **b** Thickness map of 3R-MoS_2 where spatial positions for the comparison with the theoretical model are marked. **c** SHG map of 3R-MoS_2 crystal with marked sampling positions for comparison with the theoretical model. **d** Theoretically expected SHG efficiency [6] compared to the measured SHG efficiency from the 3R-MoS_2 sample used in this work. The sample reaches about 3 % of the theoretical maximum generation efficiency.

Next, we measure an SHG map of our sample for excitation at the degenerate SPDC wavelength $2 \times \lambda_p = 1576$ nm, see Fig. 1(e) of the main manuscript and Supplementary Fig. 5(c). This wavelength is chosen to correspond to the reverse process of degenerate SPDC excited with a pump laser at $\lambda_p = 788$ nm. We then sample the SHG efficiency at various positions with different crystal thicknesses t, see Fig.5(b), and fit the SHG efficiency to the theoretical model for SHG in thin-films developed in [6]. For the calculation of the theoretical SHG curve, we use for 3R-MoS_2 the refractive index $n(\lambda = 1576\,\text{nm}) = 3.92$ at the fundamental wavelength and the index $n(\lambda = 788\,\text{nm}) = 4.39$ at the second-harmonic wavelength [6] and for quartz the index $n = 1.44$. The dependence of the SHG efficiency on the sample thickness t agrees well with the theoretical prediction. From there we conclude, that our sample with a thickness of $t = 285$ nm reaches about 3 % of the theoretical maximum SHG efficiency, which is reached at $t \approx 800$ nm. This is close to the coherence length L_c of $L_c \approx 840$ nm for SHG pumped at 1576 nm. Due to the quantum-classical correspondence between SHG and degnerate SPDC, it is expected that also the photon-pair generation would increase by a factor of $\approx 33\times$ for a sample with $t \approx 800$ nm. This shows, that the SPDC rate from 3R-MoS_2 can be straightforwardly improved with optimized sample geometries.

SUPPLEMENTARY NOTE 4: DETAILS OF EXPERIMENTAL SETUP FOR PHOTON-PAIR MEASUREMENTS

The schematics of the experimental setups as shown in Fig. 2(a) and Fig. 4(a), respectively, show only the components that are relevant to the investigated physics of the experiment. In supplementary figures 6 and 7, respectively, the full set of optical components is marked. In particular, the imaging arm with the camera used for imaging the sample for finding the measurement position as well as measurement of SHG intensities is shown.

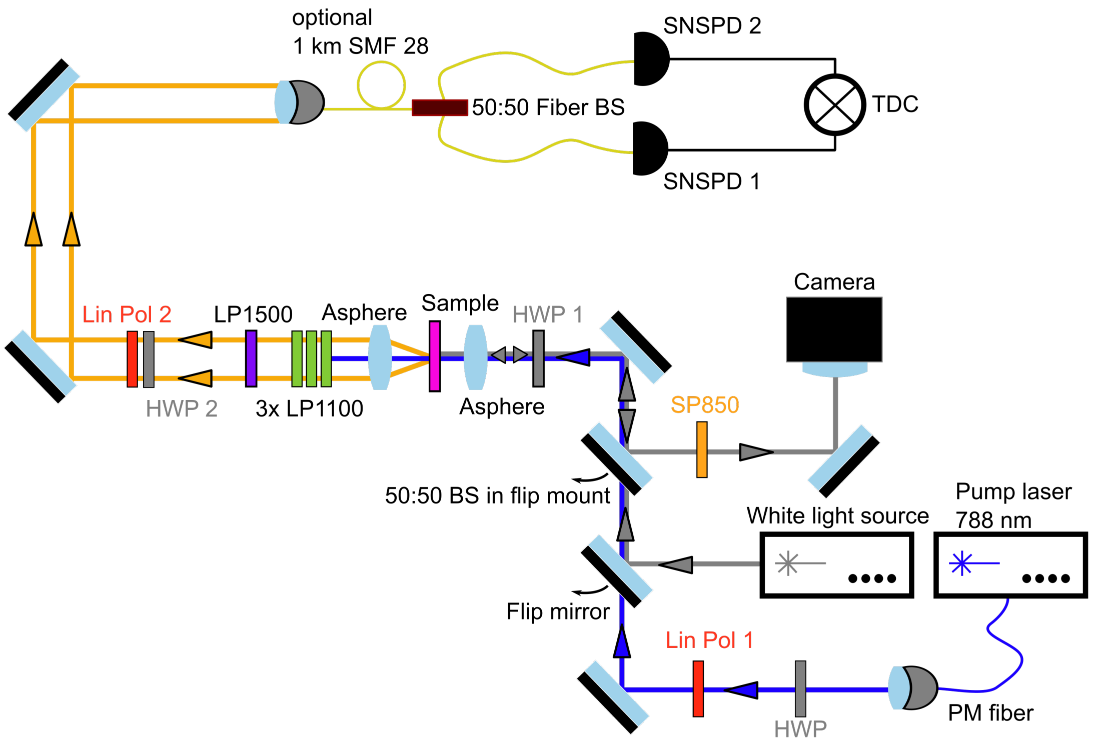

Supplementary Fig. 6. **Detailed schematic of experimental setup for correlation measurements with one coupling port and fiber beam-splitter.** Used abbreviations: PM-fiber - polarization maintaining fiber, HWP - half-wave plate, Lin Pol - linear polarizer, SP850 - short-pass filter with cut-off wavelength 850 nm, LPXXXX - long-pass filter with cut-on wavelength XXXX, SMF - single-mode fiber, BS - beamsplitter, SNSPD - superconducting nanowire single-photon detector, TDC - time-to-digital converter. Blue lines: pump beam-path, orange line: signal/idler beam-path, gray lines: beampath of white light source and image from sample, respectively. Arrows denote the propagation direction.

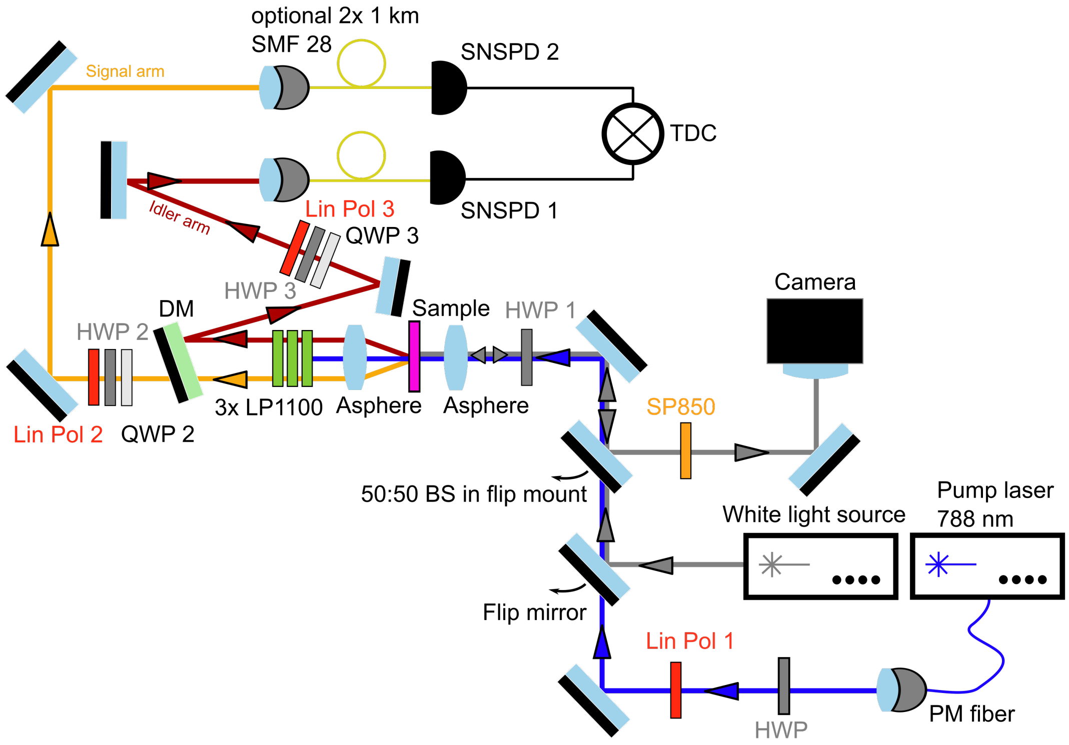

Supplementary Fig. 7. **Detailed schematic of experimental setup for correlation measurements with one coupling port and dichroic mirror.** Used abbreviations: PM-fiber - polarization maintaining fiber, DM - Dichroic mirror, HWP - half-wave plate, Lin Pol - linear polarizer, SP850 - short-pass filter with cut-off wavelength 850 nm, LPXXXX - long-pass filter with cut-on wavelength XXXX, SMF - single-mode fiber, BS - beamsplitter, SNSPD - superconducting nanowire single-photon detector, TDC - time-to-digital converter. Blue lines: pump beam-path, orange line: signal/idler beam-path, gray lines: beampath of white light source and image from sample, respectively. Arrows denote the propagation direction.

SUPPLEMENTARY NOTE 5: RAW DATA COINCIDENCE MEASUREMENTS

A. Power Dependence Test

The raw data underpinning the power dependence plot in the inset of Fig. 2(c) of the main text is shown in Supplementary Fig. 8. The histograms are measured using the experimental setup outlined in Fig. 2(a) of the main text and Supplementary Fig. 6 with a bin-width of $\tau_{\text{bin}} = 810\,\text{ps}$ and using an integration time of $t = 10\,\text{min}$. Note that the power values refer to the power incident on the sample. The high refractive index of 3R-MoS_2 at the pump wavelength $n(788\,\text{nm}) = 4.39$ [6] leads to considerable reflection, reducing the pump power effectively interacting with the nonlinear medium in the experiment. The coincidence-to-accidental ratio (CAR) follows from the ratio of SPDC rate R_{SPDC} to the accidental coincidence rate R_{acc}. The SPDC rate is the measured total coincidence rate R_{c} corrected for accidental coincidences $R_{\text{SPDC}} = R_{\text{c}} - R_{\text{acc}}$. A very related quantity is the normalized second-order correlation function at zero time delay $g^{(2)}(0)$ which can be measured as $g^{(2)}(0) = R_{\text{c}}/R_{\text{acc}}$. Therefore we have in total the relation [13]

$$\text{CAR} = \frac{R_{\text{SPDC}}}{R_{\text{acc}}} = \frac{R_{\text{c}} - R_{\text{acc}}}{R_{\text{acc}}} = g^{(2)}(0) - 1. \tag{15}$$

The measured CAR calculated from each of the histograms is shown in Supplementary Fig. 9. Note that the CAR for the input power of $P = 17.2\,\text{mW}$ is computed based on the histogram shown in Fig. 2(c) of the main text. The longer integration time of 3.5 h in this case leads to a reduced uncertainty, compared to the other data points.

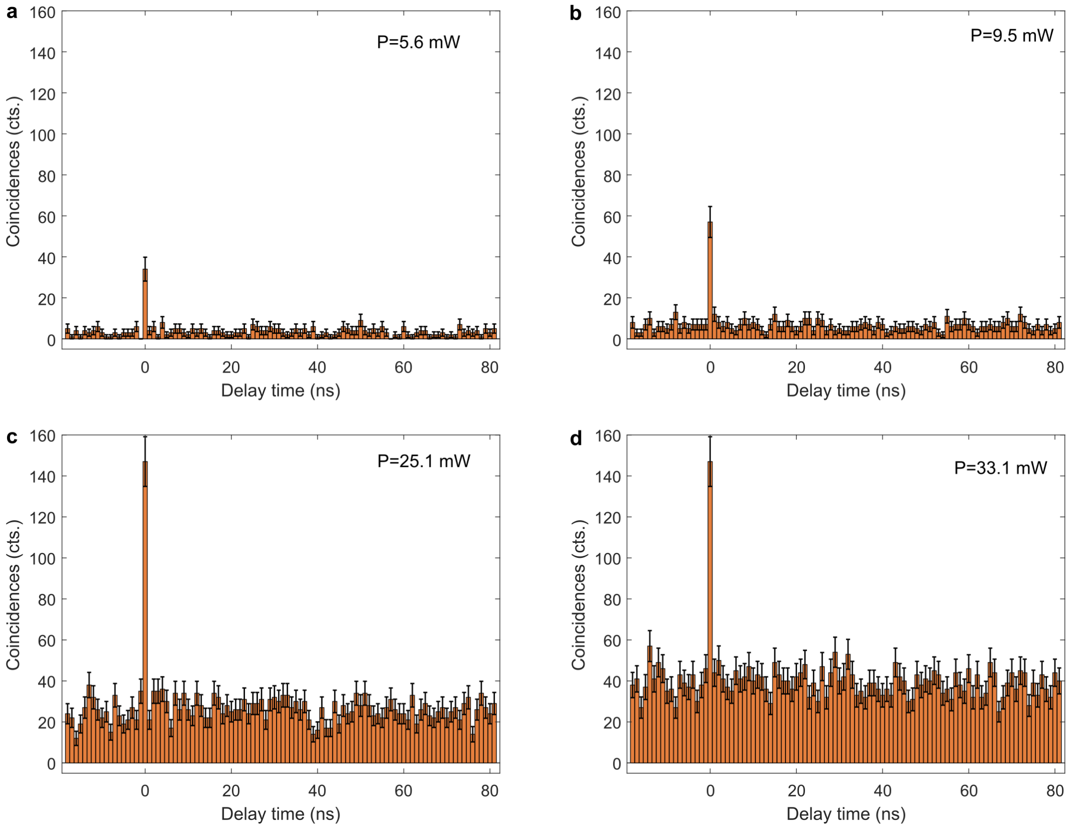

Supplementary Fig. 8. Coincidence histograms for different excitation powers. Coincidence histograms recorded for excitation powers (a) 5.6 mW, (b) 9.5 mW, (c) 25.1 mW and (d) 33.1 mW for an integration time of 10 min.

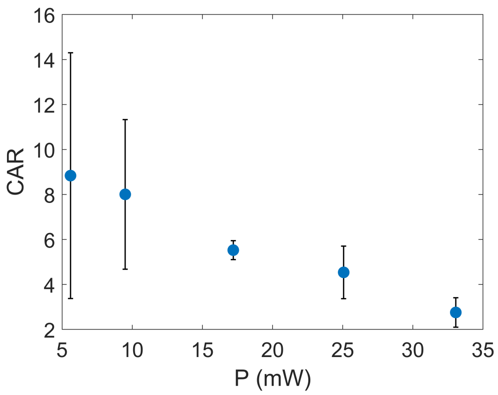

Supplementary Fig. 9. **Coincidence-to-accidental ratio (CAR) for varying pump powers.** CAR calculated from the coincidence histograms at different power levels shown in Supplementary Fig. 8. Note that the CAR for power $P = 17.2\,\mathrm{mW}$ is computed based on the histogram shown in Fig. 2(c) of the main text.

B. Polarization Analysis

Figures 10 and 11 contain the raw coincidence histograms which underlay the angularly resolved SPDC plots shown in Fig. 3 of the main text. The experiments are carried out using the experimental setup shown in Fig. 2(a) of the main text and Supplementary Fig. 6. In all measurements, the integration time is $t = 20\,\mathrm{min}$ for each histogram. The bin-width of the histogram is $\tau_{\mathrm{bin}} = 150\,\mathrm{ps}$, the excitation laser power is $P = 17.2\,\mathrm{mW}$. The pump polarization direction was controlled by a rotating half-wave plate before the pump focusing lens. The rotating linear polarizer is practically realized using a rotating half-wave plate in front of a fixed linear polarizer. This configuration is chosen in order to keep the detection polarization direction for the superconducting nanowire single-photon detectors (SNSPD) constant, since this detector type does show a pronounced polarization dependence in its detection efficiency[14]. Note therefore, that in the case where no detection direction is defined Supplementary Fig. 10(a) the polarization dependence of the SNSPDs can lead to a residual modulation of the detected pair-rate R_{SPDC} which in general should be constant. The maximum rate for detection through a linear polarizer should be half of the maximum rate for detection without a polarizer. As shown in Supplementary Fig. 10 and Supplementary Fig. 11, this relation holds well but not perfectly for the experimental data. For practical reasons, in our the SNSPDs and the remaining experimental setup are physically located in different rooms and are connected by 70 m long, non-polarization maintaining single-mode fibers. These fibers are guided through non-temperature controlled parts of the building. Small temperature drifts which affect the output polarization state after the long, non-polarization maintaining single-mode fibers between the different days of measurement may therefore have slightly changed the effective efficiency of the detection system.

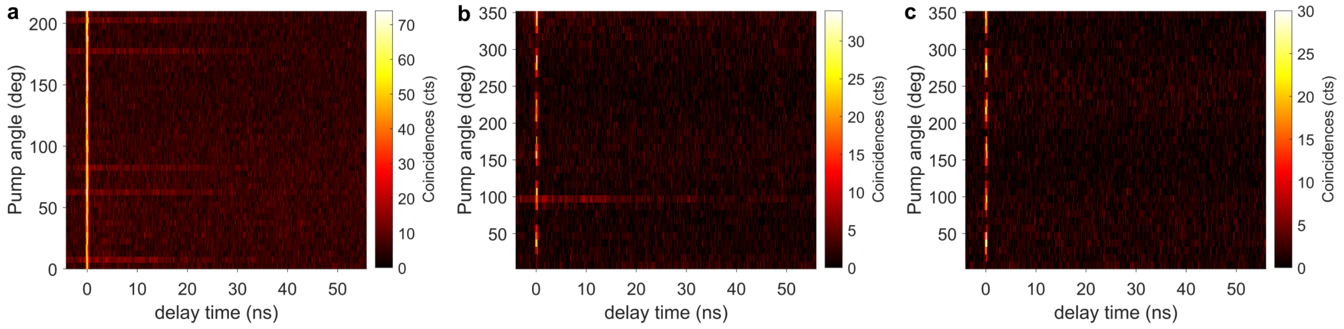

Supplementary Fig. 10. **Coincidence histograms for rotating SPDC pump and unpolarized detection or detection with rotating polarizer.** For all measurements, the integration time is $t = 20\,\mathrm{min}$ for each pump polarization angle. The bin-width of the histogram is $\tau_{bin} = 150\,\mathrm{ps}$, the excitation laser power is $P = 17.2\,\mathrm{mW}$. **a** Coincidence histograms for rotating pump polarization and detection of all SPDC polarization directions (raw data for Fig. 3(b) of the main text). **b,c** Coincidence histograms for rotating pump polarization and detection of photon-pairs through linear polarizer aligned collinearly (b) or perpendicularly (c) to the pump polarization (raw data for Fig. 3(c) of the main text).

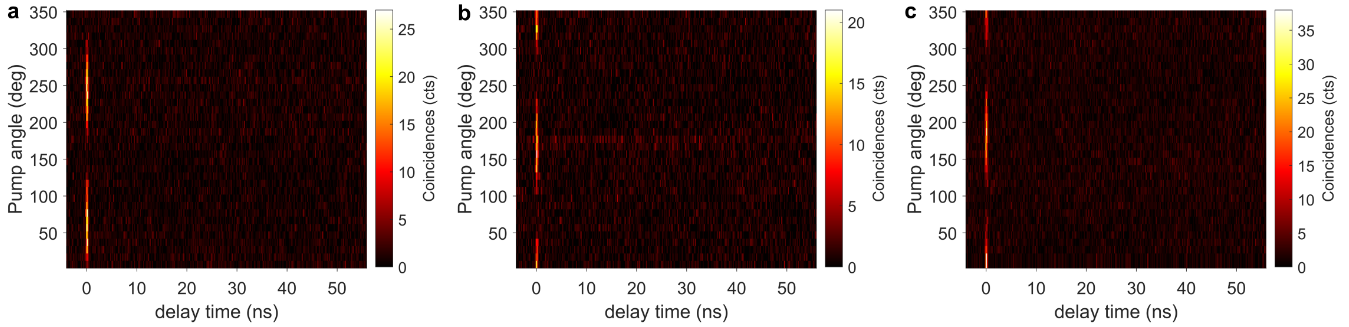

Supplementary Fig. 11. **Coincidence histograms for rotating SPDC pump and detection through fixed polarizer.** For all measurements, the integration time is $t = 20\,\mathrm{min}$ for each pump polarization angle. The bin-width of the histogram is $\tau_{bin} = 150\,\mathrm{ps}$, the excitation laser power is $P = 17.2\,\mathrm{mW}$. **a-c** Coincidence histograms for rotating pump polarization and detection through fixed polarizer oriented at (a) 110° (20° offset from AC-axis), (b) 55° (25° offset from AC-axis) and (c) 50° (20° offset from AC-axis). Raw data for Fig. 3(b)-(f) of the main text.

C. Quantum-State Tomography

For the quantum-state tomography, we use the experimental setup sketched in Fig. 4(a) of the main text, where we subsequently project the two-photon state into the following bases: $|\mathrm{HH}\rangle$, $|\mathrm{HV}\rangle$, $|\mathrm{VV}\rangle$, $|\mathrm{VH}\rangle$, $|\mathrm{RH}\rangle$, $|\mathrm{RV}\rangle$, $|\mathrm{DV}\rangle$, $|\mathrm{DH}\rangle$, $|\mathrm{DL}\rangle$, $|\mathrm{DD}\rangle$, $|\mathrm{RD}\rangle$, $|\mathrm{HD}\rangle$, $|\mathrm{VD}\rangle$, $|\mathrm{VR}\rangle$, $|\mathrm{HR}\rangle$, $|\mathrm{RR}\rangle$. In Supplementary Fig. 12, the number of coincidence counts obtained in each basis for the two different excitation configurations y-polarized and x-polarized are shown. The number of coincidences is extracted from the coincidence histograms shown in Supplementary Fig. 13 and Supplementary Fig. 14, respectively, by summing over ± 2 time bins next to the zero-delay bin and subtracting the averaged number of accidentals. The bin-width of the histograms is $\tau_{\mathrm{bin}} = 300\,\mathrm{ps}$ and the integration time is $t = 30\,\mathrm{min}$ at a pump power of $P = 63\,\mathrm{mW}$. Based on these extracted count-rates, the quantum state is reconstructed using an established code based on maximum likelihood estimation [2], which ensures the retrieval of a physical density matrix despite the presence of noise in the experiment. As a starting point for the maximum likelihood estimation, we use a linear tomography on the measurement data [2]. The fidelity of the retrieved density matrix with different reference states is calculated using Eq. (7).

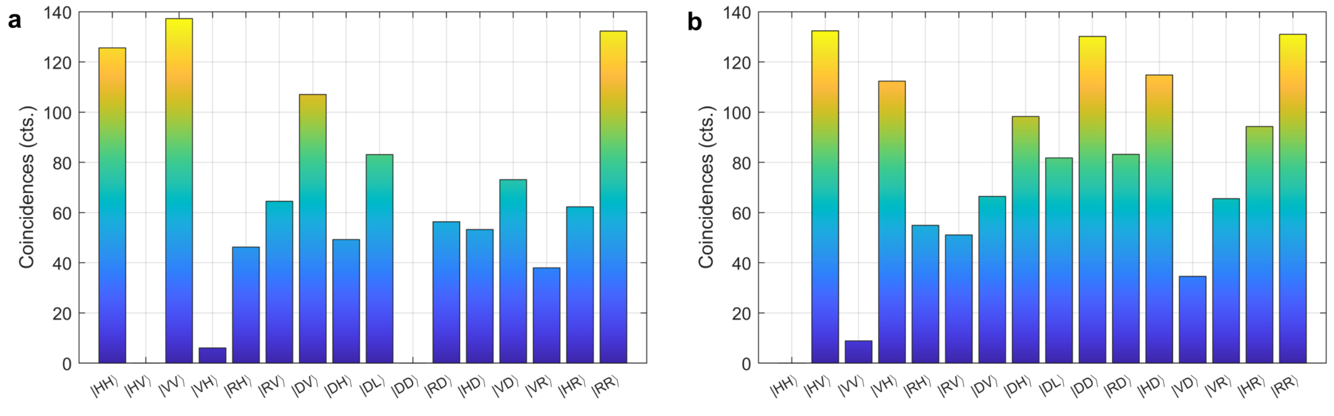

Supplementary Fig. 12. **Coincidence counts of quantum state tomography** Coincidence counts after accidental subtraction in 16 different bases for tomographic reconstruction of two-photon quantum state for (a) y-polarized excitation and (b) x-polarized excitation. The coincidence counts are extracted from the histograms in Supplementary Fig. 13 and 14, respectively.

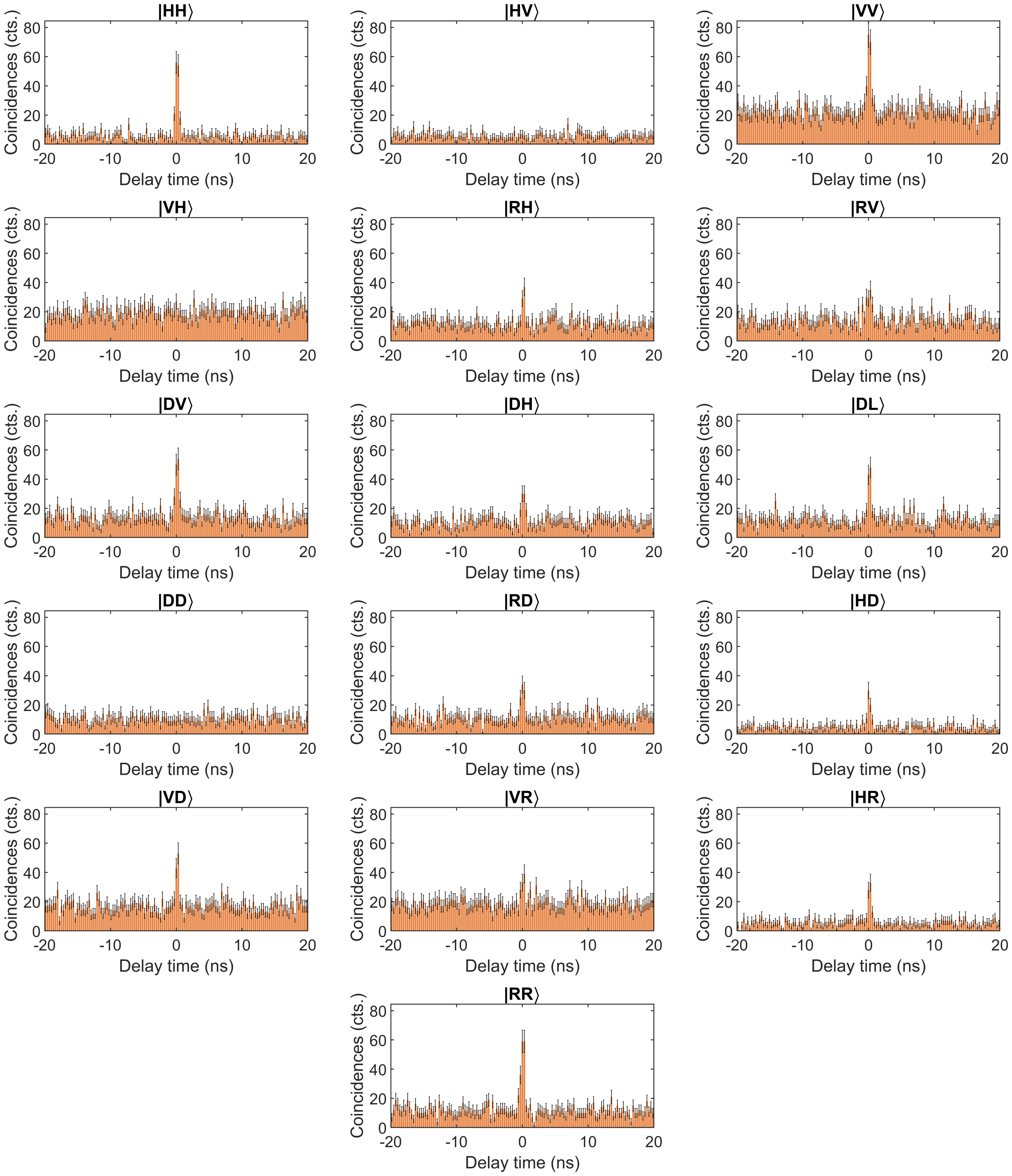

Supplementary Fig. 13. **Coincidence histograms of quantum state tomography for** y**-polarized excitation.** Histograms of coincidence measurements in 16 different bases for tomographic reconstruction of two-photon quantum state for y-polarized excitation.

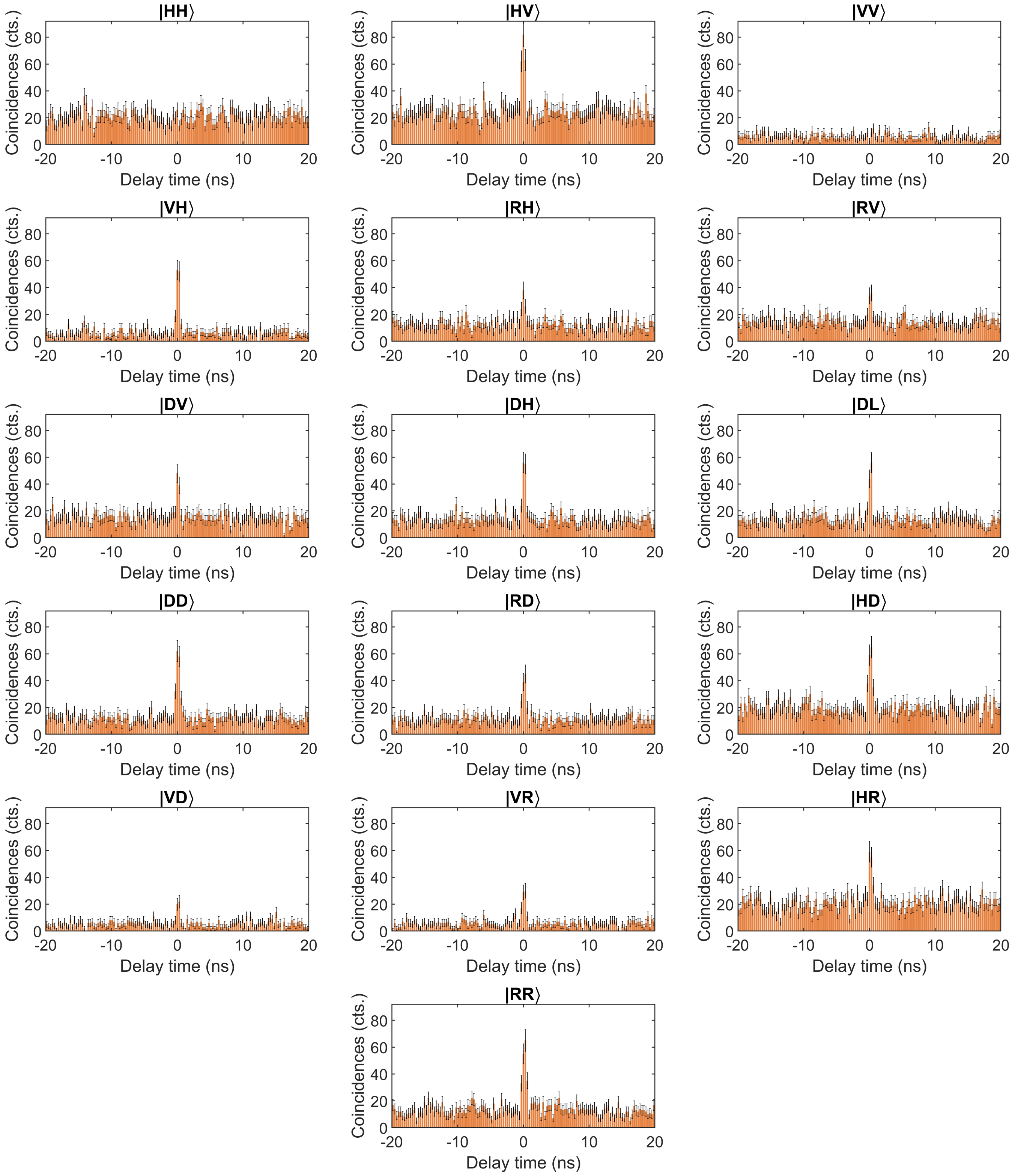

Supplementary Fig. 14. Coincidence histograms of quantum state tomography for x-polarized excitation. Histograms of coincidence measurements in 16 different bases for tomographic reconstruction of two-photon quantum state for x-polarized excitation.

REFERENCES

[1] Wootters, W. K. Entanglement of Formation of an Arbitrary State of Two Qubits. *Physical Review Letters* **80**, 2245–2248 (1998).

[2] James, D. F. V., Kwiat, P. G., Munro, W. J. & White, A. G. Measurement of qubits. *Physical Review A* **64**, 052312 (2001).

[3] Nielsen, M. A. & Chuang, I. L. *Quantum Computation and Quantum Information: 10th Anniversary Edition* (Cambridge University Press, 2010).

[4] Li, Y. *et al.* Probing Symmetry Properties of Few-Layer MoS2 and h-BN by Optical Second-Harmonic Generation. *Nano Letters* **13**, 3329–3333 (2013).

[5] Boyd, R. W. *Nonlinear Optics* (Academic Press, 2008), 3rd edn.

[6] Xu, X. *et al.* Towards compact phase-matched and waveguided nonlinear optics in atomically layered semiconductors. *Nature Photonics* **16**, 698–706 (2022).

[7] Poddubny, A. N., Iorsh, I. V. & Sukhorukov, A. A. Generation of photon-plasmon quantum states in nonlinear hyperbolic metamaterials. *Physical Review Letters* **117**, 123901 (2016).

[8] Weissflog, M. A. *et al.* Nonlinear nanoresonators for Bell state generation. *Applied Physics Reviews* **11**, 011403 (2024).

[9] Santos, E. A., Pertsch, T., Setzpfandt, F. & Saravi, S. Subdiffraction quantum imaging with undetected photons. *Phys. Rev. Lett.* **128**, 173601 (2022).

[10] Santos, E. A., Weissflog, M. A., Pertsch, T., Setzpfandt, F. & Saravi, S. Entangled photon-pair generation in nonlinear thin-films. *Nanophotonics* (2024).

[11] Wagoner, G. A., Persans, P. D., Van Wagenen, E. A. & Korenowski, G. M. Second-harmonic generation in molybdenum disulfide. *Journal of the Optical Society of America B* **15**, 1017 (1998).

[12] Shi, J. *et al.* 3R MoS2 with Broken Inversion Symmetry: A Promising Ultrathin Nonlinear Optical Device. *Advanced Materials* **29**, 1701486 (2017).

[13] Okoth, C., Cavanna, A., Santiago-Cruz, T. & Chekhova, M. V. Microscale Generation of Entangled Photons without Momentum Conservation. *Physical Review Letters* **123**, 263602 (2019).

[14] Chang, J. *et al.* Multimode-fiber-coupled superconducting nanowire single-photon detectors with high detection efficiency and time resolution. *Applied Optics* **58**, 9803–9807 (2019).
